# Renal Abscess Caused by Extended-Spectrum Beta-Lactamase-Producing Bacteria and Complicated by the Perforation to a Cyst and to the Renal Pelvis

**DOI:** 10.1089/cren.2016.0022

**Published:** 2016-06-01

**Authors:** Jan Novak, Viktor Vik, Roman Zachoval, Truls Erik Bjerklund Johansen

**Affiliations:** ^1^ Department of Urology, Thomayer Hospital, Prague, Czech Republic.; ^2^ Department of Urology, Oslo University Hospital and Institute of Clinical Medicine, University of Oslo, Norway.

## Abstract

We report a 50-year-old female patient with a left-sided renal abscess caused by extended-spectrum β-lactamase-producing bacteria. According to the ORENUC classification she had phenotype N. The course was complicated by a perforation to an adjacent cyst and later to the renal pelvis. A primarily conservative approach of intravenous antibiotics had to be changed to an ultrasonography-guided percutaneous drainage of the lesion and insertion of a ureteral stent to stem a high volume of urine leakage. Drainage of a renal abscess is indicated if the size is larger than 3 cm according to EAU guidelines (relative size) or when the resolution does not occur after antibiotics. One-year follow-up showed the patient made a full recovery with no recurrence of a urinary tract infection or of any abscess.

## Introduction and Background

Renal abscesses most commonly occur as a complication of a urinary tract infection (UTI). However, they may also be caused by bacteriemia from nonurological infections. Complications of UTIs are usually associated with one or more risk factors (RFs). EAU stratifies RFs into six categories using ORENUC phenotyping (n**O** known/associated RF; **R**ecurrent UTI RF; **E**xtra-urogenital RF; **N**ephrological RF; **U**rological RF, which can be resolved during therapy; permanent urinary **C**atheter and nonresolvable urological RF). According to this classification, our patient had a nephrological RF or phenotype N. Sometimes no RFs are found and the patient has phenotype O.^1,2^

A formation of microabscesses is common in acute febrile pyelonephritis with severity grade 3. Microabscesses probably occur as a result of parenchymal edema and focal ischemia, and usually resolve with antibiotic treatment.^1^ Renal abscesses located within the kidney capsule may rupture externally and lead to a perinephric, paranephric, or even psoas abscess. Rupture to the urinary system may also be evident.

In the past, most renal abscesses were caused by *Stahylococcus* species arising from infected skin lesions in patients with compromised immune systems, that is, diabetics, patients on hemodialysis, and intravenous drug abusers. Due to better management of underlying diseases and more effective antibiotic treatment of skin lesions, abscesses caused by *Enterobacteriaceae* species have become recently more frequent.^3^

Abscesses of hematogenous origin are frequently located in the renal cortex.^3^ This is probably related to the fact that 90% of the renal perfusion goes through the cortex. Abscesses caused by *Enterobacteriaceae* species are frequently located in the corticomedullary junction and are very often seen in patients with phenotypes E and U RFs.^3^

## Case Presentation

A 50-year-old female was referred by an office urologist to our department for suspected left-sided renal abscess based on clinical and ultrasonographic findings. She complained of left-sided flank pain, shivering, and dizziness and had fever of 38.0°C (100.4°F). She had been diagnosed with autoimmune thyroiditis ∼20 years ago. She also had an allergy to iodinated contrast media but was otherwise fit and well with no history of recurrent UTIs. She was currently not receiving any treatment.

**FIG. 1.**
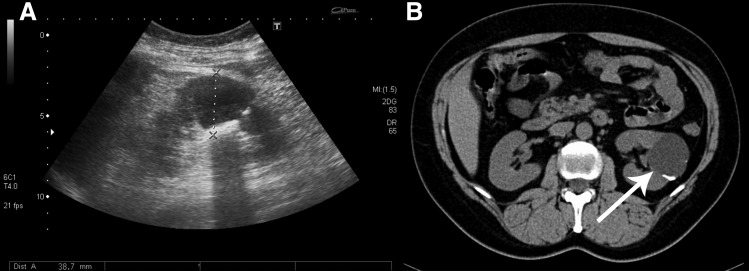
Complicated cyst diameter 41 mm, Bosniak IIF, calcifications in the wall by ultrasonography **(A)**, diagnosed 10 years ago by noncontrast CT **(B)**, no signs of progression.

Noncontrast CT performed a decade ago revealed a complicated cyst measuring 41 mm in diameter with a wall calcification, classified as Bosniak IIF. The cyst had been followed up by ultrasonography with no signs of progression (Fig. 1).

Blood chemistry showed significantly elevated inflammatory markers (c-reactive protein [CRP] 302.3 mg/L, white blood cell count [WBC] 18.7 × 10^9^/L) and mild renal function alteration with serum creatinine 146 μmol/L and urea 4.4 mmol/L. Urine microscopy showed WBCs. Urine culture was taken on admission.

**FIG. 2.**
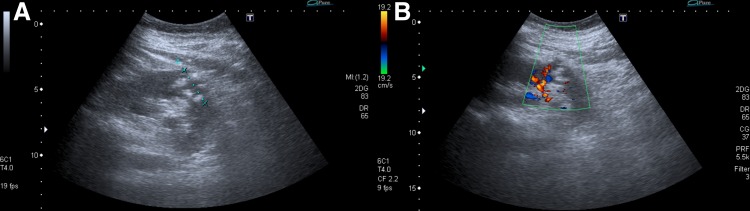
Renal abscess located dorsally and caudally from the cyst, diameter 29 mm, hypoechoic content with floating particles **(A)**, increased perfusion around **(B)**, by ultrasonography.

Ultrasonography revealed a new cystic lesion in the left kidney measuring 29 mm in diameter with hypoechoic content of floating particles and increased perfusion around (Fig. 2). The distance between the pre-existing cyst and the new lesion was ∼1 cm. No pathology was found on the right kidney.

A conservative approach was decided and the patient was empirically provided with gentamicin 160 mg i.v. q.d. (dose reduction due to renal function impairment) in combination with co-amoxicillin 1.2 g i.v. t.i.d.

Two days later, the results of mid-stream sample of urine culture showed *Enterobacter* sp. 10^3^ cfu/mL—resistant to ampicillin, cefalotin, co-amoxicillin, cefuroxime, and susceptible to trimethoprim/sulfamethoxazole, gentamicin, ofloxacin, nitrofurantoin, norfloxacin, tetracycline, cefotaxime, and ceftazidime. Co-amoxicillin was stopped because of resistance and was replaced by ofloxacin 200 mg p.o. b.i.d. Gentamicin was given for further 5 days. Serum inflammatory markers and renal function tests showed improvement after treatment (CRP 101.0 mg/L, WBC 11.0 × 10^9^/L, creatinine 68 μmol/L, urea 2.8 mmol/L), and subjective assessment of health by the patient was positive.

**FIG. 3.**
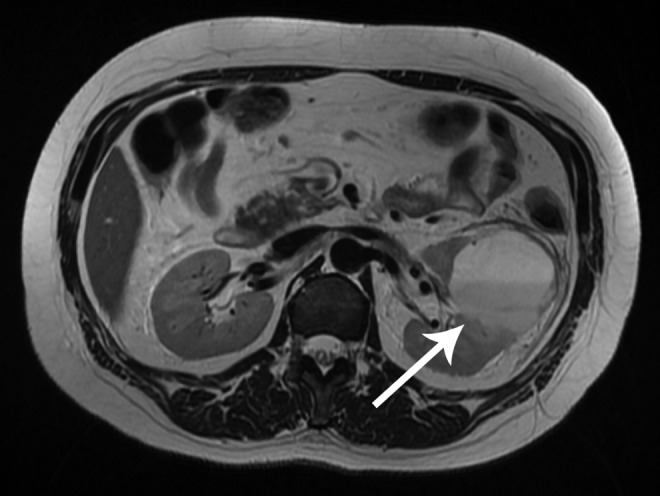
Suspect protrusion of the abscess to the cyst by MRI.

**FIG. 4.**
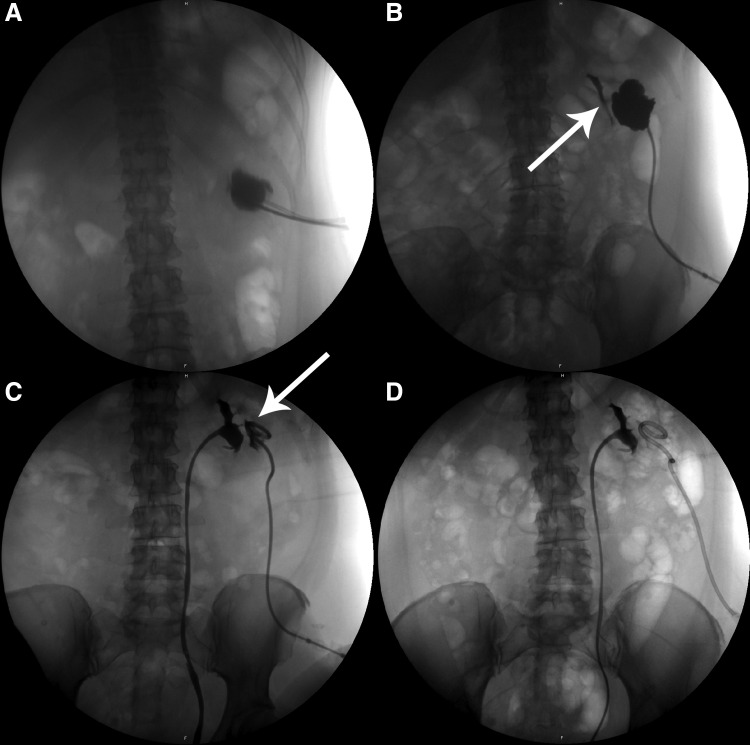
Percutaneous insertion of two drains into the abscess **(A)**, filiform leakage of contrast to the urinary system **(B)**, insertion of a new Single-J stent; persistent communication between urinary system and abscess **(C)**, no more signs of communication between urinary tract and abscess **(D)**.

Control imaging with MRI raised suspicion that the abscess had perforated to the adjacent cyst. Stratification of liquid contents with different intensity suggested to represent pus inside the lesion (Fig. 3). We therefore inserted two percutaneous drains into the lesion (Fig. 4A) for optimal drainage and 70 mL of pus was evacuated. One drain dislocated the following day. Cultures showed the same bacterium as had been found in the urine.

The amount of drained fluid increased dramatically after 1 week postdrain from 55 to 700 mL a day. The creatinine concentration in the drained fluid was 2627 μmol/L, clearly showing that the fluid was urine. Intravenous iodinated contrast medium was injected through the drain, which revealed a filiform communication to the urinary system. A Double-J stent (6F) was inserted immediately and was followed by a prompt reduction in the external drainage fluid to 10 mL per day (Fig. 4B).

However, the drained volume increased again to 650 mL/day on the 12th day after admission. We have now replaced the Double-J stent with a long Single-J stent to provide better monitoring of the total urine production of the left kidney (Fig. 4C).

We further performed retrograde pyelography on the 15th day, which showed no signs of communication between the urinary system and the lesion (Fig. 4D) and the external drain was removed. The Single-J stent was shortened so that the lower end was in the bladder. The patient was discharged 17 days after admission and kept a bladder catheter for 24 days.

Two months later, ultrasonography again showed a complicated cyst measuring 48 mm in diameter but no signs of an abscess. The patient's private urologist was recommended to remove the Single-J stent after 3 months. At 1-year recall, the patient had no recurrence of UTIs or abscesses and ultrasonography has not showed any changes in the renal cyst.

## Discussion

Although clinical and laboratory findings are indicative, imaging is a prerequisite for diagnosing a renal abscess. Ultrasonography is the first choice modality, but contrast CT or MRI is necessary for confirmation of an abscess and exclusion of a concomitant malignant tumor.

UTIs of high severity grade in patients with urological (ORENUC phenotype U), nephrological (N), and extra-urogenital (E) RFs and especially in case of hospital-acquired infections are caused by a wide spectrum of bacterial strains, often with a broad resistance pattern. Alternative antibiotics for empirical treatment are fluoroquinolones, amino-penicillines plus β-lactam inhibitors, third-generation cephalosporins, or aminoglycosides. The choice of antibiotics depends on the local resistance pattern and the patient's history. Most of these antibiotics require intravenous administration. Combination of antibiotics is strongly advised in case of urosepsis. The empirical treatment should be adapted according to the results of culture as soon as they are available.

According to the EAU guidelines, a conservative approach with antibiotic treatment is only indicated in abscesses with a diameter up to 3 cm. Larger lesions should be drained percutaneously. In certain cases, open surgery or nephrectomy is the only treatment option.^2^ In our opinion, the present patient was managed according to EAU guidelines and the external drainage was performed as soon as the perforation was diagnosed. Some authors recommend drainage in case of persistent fever and no change of the abscess on imaging after antibiotic treatment for several days.^2^

A solitary cyst is not a RF for development of complicated UTI and is not a reason for modifying standard treatment of a renal abscess. However, cysts causing pelvic obstruction may require puncture and drainage. Patients with multiple cysts as part of autosomal dominant polycystic kidney disease often have renal insufficiency or ORENUC phenotype N, which means increased risk of a more severe outcome. EAU guidelines do not consider neighboring cysts. In our opinion, a perforation to near-by cysts should be kept in mind in cases of delayed defervescence.

As demonstrated in our patient, a cutaneous fistula from the renal pelvis through an infected cyst requires adequate drainage of the renal pelvis and a lot of patience by the urologist. Finally, we believe RFs should be described according to EAU ORENUC phenotyping in patients with complicated UTIs.

## Abbreviations Used

b.i.d.: bis in die (in Latin, two times a day)
CRP: c-reactive protein
EAU: European Association of Urology
i.v.: intravenous
p.o.: per os (in Latin, orally)
q.d.: quaque die (in Latin, once a day)
RF: risk factor
t.i.d.: ter in die (in Latin, three times a day)
UTI: urinary tract infection
WBC: white blood cell count
